# Brachial Plexopathies: A Comprehensive Radiologic Method Integrating Ultrasound and MRI

**DOI:** 10.3390/jcm14176311

**Published:** 2025-09-06

**Authors:** Giulia Pacella, Raffaele Natella, Federico Bruno, Michela Bruno, Donatella Franco, Daniele Giuseppe Romano, Marcello Zappia

**Affiliations:** 1 Department of Medicine and Health Science “V. Tiberio”, University of Molise, 86100 Campobasso, Italy; raffaele.natella@unimol.it (R.N.); m.bruno10@studenti.unimol.it (M.B.); marcello.zappia@unimol.it (M.Z.); 2 Fondazione Trotula de Ruggiero, 84121 Salerno, Italy; 3 Ospedale S. Salvatore, 67100 L’Aquila, Italy; federico.bruno.1988@gmail.com; 4 Department of Sperimental Medicine, Università degli Studi della Campania Luigi Vanvitelli, 80138 Napoli, Italy; d.franco1@studenti.unimol.it; 5 Unit of Diagnostic and Interventional Neuroradiology, 84131 Salerno, Italy; daniele.romano@sangiovannieruggi.it

**Keywords:** brachial plexus, plexopathy, traumatic plexopathy, neoplastic plexopathy, ultrasound, magnetic resonance imaging, MR neurography, diffusion tensor imaging

## Abstract

**Background:** Brachial plexopathies comprise a diverse array of illnesses with multifactorial etiologies, including trauma, inflammation, neoplasia, and iatrogenic damage, frequently manifesting with nonspecific clinical symptoms. Precise and prompt imaging evaluation is essential for diagnosis, treatment planning, and monitoring. **Objective:** To equip radiologists with interpretative tools for a systematic assessment of the brachial plexus utilizing advanced imaging modalities, specifically ultrasound (US) and magnetic resonance imaging (MRI), while emphasizing techniques, indications, limitations, and critical radiologic signs for differential diagnosis. **Imaging Techniques:** This narrative review concentrates on US and MRI. High-frequency linear probes with multiplanar dynamic scans provide US visualization of trunks, cords, and terminal branches in superficial areas. Specialized MRI procedures (T1, T2, STIR, DWI, contrast-enhanced) provide comprehensive evaluation of spinal roots and deep tissues, differentiating preganglionic from postganglionic lesions. A combined US–MRI methodology can enhance diagnostic efficacy. **Findings:** Ultrasound is excellent for superficial and dynamic assessment, especially in post-traumatic and iatrogenic lesions, while MRI is the gold standard for deep structures and complex disorders. The integration of two modalities enhances lesion identification and treatment direction. Emerging methodologies further enhance diagnostic and prognostic capabilities. **Conclusions:** The synergistic application of US and MRI, emphasizing nerve injury patterns and muscle denervation indicators, facilitates precise and prompt diagnosis of brachial plexopathies. Standardizing imaging standards and incorporating modern techniques are essential for interdisciplinary, customized patient care.

## 1. Introduction

The brachial plexus is a complicated anatomical structure that extends from the cervical spine to the axilla, supplying motor, sensory, and autonomic innervation to the upper limb. The anatomy is segmented into five nerve components: roots, trunks, divisions, cords, and terminal branches, which are variably organized and located deep inside the neck and shoulder girdle. The complex structure, together with anatomical variations and overlapping symptoms with cervical radiculopathy, makes diagnosing brachial plexopathies especially difficult in both traumatic and non-traumatic situations [1].

Brachial plexus problems may exhibit nonspecific symptoms, including pain, paresthesia, and weakness, which may resemble other neurological or musculoskeletal conditions. Trauma is the predominant etiology, succeeded by neoplastic infiltration; less prevalent causes encompass radiation-induced, autoimmune, and infectious neuropathies. Given the plexus’s deep anatomical positioning and the diverse array of potential pathologies, imaging is essential for diagnosis, treatment planning, and surgical navigation. In traumatic plexopathy, there are specific imaging findings and therapy strategies for pre- versus postganglionic injuries; nevertheless, for nontraumatic plexopathies, obtaining a precise patient history is sometimes essential [2].

MRI has always served as the benchmark for assessing brachial plexus pathology, owing to its multiplanar capabilities, exceptional soft-tissue contrast, and proficiency in identifying both direct nerve signal anomalies and indirect indicators such as muscle denervation. Recently, MR Neurography has become the ideal technique for the thorough evaluation of severe brachial plexus injuries. Contemporary MRI Neurography techniques utilize high-resolution 2D and 3D sequences (e.g., STIR, fat-suppressed T1, and proton density), facilitating precise assessment of nerve root continuity, alterations in signal intensity, and participation of the postganglionic segment. Importantly, neurography differentiates between Sunderland grade IV NIC (neuroma-in-continuity) and grade V (total transection) injuries and frequently surpasses CT myelography in evaluating postganglionic lesions. Advanced methodologies, including Dixon-based fat suppression and 3D MIP reconstructions, significantly improve visibility, especially in the retro- and infraclavicular areas.

Concurrently, US has emerged as a formidable and accessible instrument for the real-time assessment of peripheral nerves, encompassing the majority of the brachial plexus segments. Acoustic shadowing imposes limitations in the retroclavicular region; nevertheless, ultrasonography provides superior spatial resolution in the supraclavicular and infraclavicular compartments, facilitating dynamic evaluation of nerve integrity, fascicular architecture, and adjacent soft tissues. Its value is especially apparent in traumatic, inflammatory, and iatrogenic plexopathies, as well as in circumstances where MRI is contraindicated or inaccessible [3].

In light of this expanding landscape, there is a growing necessity for radiologists to proficiently acquire both ultrasound and MRI procedures for brachial plexus imaging. This study seeks to deliver a systematic and pragmatic guidance for the cohesive interpretation of ultrasound and MR images of the brachial plexus, emphasizing anatomical landmarks, imaging procedures, and diagnostic patterns across many clinical circumstances.

## 2. Anatomical Components

Peripheral nerves are complex structures composed of axons grouped into fascicles, which are surrounded by protective connective tissue layers: the endoneurium, perineurium, and epineurium. These layers provide structural support, mechanical resilience, and vascular supply. Schwann cells contribute to myelination, ensuring efficient nerve conduction. The vascular network and blood–nerve barrier play a key role in maintaining axonal health. A clear understanding of this anatomy is essential for interpreting both normal and pathological imaging findings. 

The brachial plexus is classically divided into five components: roots, trunks, divisions, cords, and terminal branches. It originates from the ventral rami of spinal nerves C5 to T1, with occasional contributions from C4 (prefixed) or T2 (postfixed variants). The roots pass between the anterior and middle scalene muscles within the interscalene triangle, adjacent to the subclavian artery. 

Notably, classical interscalene trunk arrangement is seen in only one-third of individuals, with two-thirds showing variants such as the superior piercing type, in which the C5–C6 roots traverse the anterior scalene muscle [4].

Three trunks are formed in the posterior cervical triangle: the superior (C5–C6), middle (C7), and inferior (C8–T1). Each trunk splits into anterior and posterior divisions, corresponding to motor function (flexors and extensors, respectively). These divisions cross the costoclavicular space, located between the clavicle and the first rib. This narrow anatomical area is often inadequately visualized on ultrasound but can be well depicted on sagittal and coronal MRI scans.

The divisions regroup to form the lateral, posterior, and medial cords, named according to their relationship to the axillary artery. The cords lie in the infraclavicular fossa, superficial to the subscapularis muscle, and are key landmarks in ultrasound-guided nerve blocks. Terminal branches include the musculocutaneous, median, ulnar, radial, and axillary nerves. Additionally, supraclavicular branches (e.g., dorsal scapular, long thoracic, and suprascapular nerves) arise from roots and trunks, while infraclavicular branches originate from the cords. Anatomical variations in the brachial plexus are common, occurring in up to 50% of individuals. These variations are most frequently seen in the supraclavicular portion of the plexus and hold significant implications for surgical planning and nerve repair procedures [2,5,6,7,8]. Table 1 summarizes the main supraclavicular anatomical variants.

## 3. Key Imaging Landmarks

Normal brachial plexus anatomy is delineated through five principal anatomical landmarks: the neural foramen, the interscalene triangle, the lateral border of the first rib, the medial border of the coracoid process, and the lateral border of the pectoralis minor muscle. These landmarks align with the anatomical subdivisions of the plexus and function as reference points that can be consistently observed using ultrasound and MRI with suitable sequences and scanning planes [6,9].

Ultrasound Landmarks: The neural foramen, originating from the C5–C8 roots, is observed laterally to the vertebral artery in an axial oblique view of the anterolateral neck.

Interscalene triangle, delineated by the anterior and middle scalene muscles; location for root/trunk visualization (“stoplight sign”).

The first rib and subclavian artery are essential for delineating divisions inside the costoclavicular space, although they are obscured by artifacts from the overlying clavicle and first rib.

The axillary artery serves as a reference point for identifying the cords within the infraclavicular fossa.

The coracoid process and pectoralis minor assist in identifying terminal branches.

**Figure 1:**
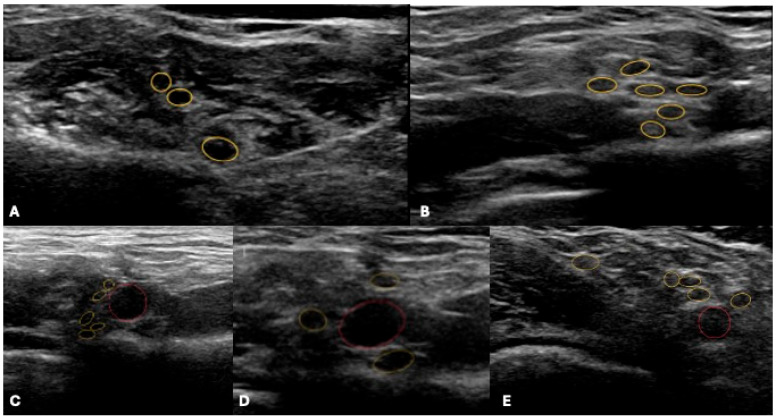
Ultrasound depiction of the brachial plexus at different anatomical levels. (**A**) Trunks (7u, lower) within the interscalene gap. (**B**) Divisions in the typical “bunch-of-grapes” configuration and (**C**) in the costoclavicular space. (**D**) Cords (posterior, medial, lateral) arranged around the subclavian artery (in red) in the retropectoralis minor space. (**E**) Terminal branches in the axillary projection: musculocutaneous (MCN), axillary (AN), radial (RN), median (MN), and ulnar (UN). Nerve structures are highlighted in yellow, and the subclavian artery in red.

Selected anatomical landmarks are illustrated in Figure 1.

**Figure 2:**
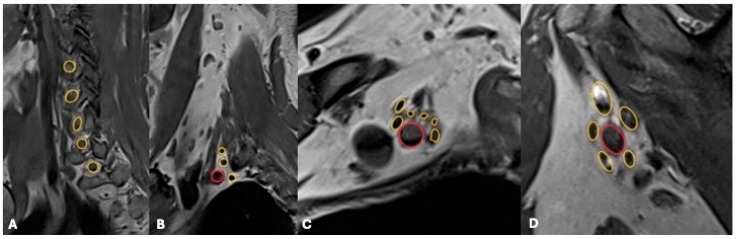
MRI of the brachial plexus in different planes and anatomical levels. Sagittal T2-weighted images depict: (**A**) cervical roots, (**B**) trunks within the interscalene triangle, (**C**) divisions surrounding the axillary artery, and (**D**) terminal branches in the axillary region (in a pathologic condition). The neuronal structures are circled in yellow, the arterial structures are circled in red.

The sagittal plane on MRI effectively demonstrates nerve continuity from roots to terminal branches, as illustrated in Figure 2.

STIR or Dixon sequences improve nerve-to-muscle contrast and emphasize edema, injury, or inflammation. Three-dimensional MIP reconstructions are essential for presurgical planning and the assessment of complex lesions [10].

## 4. Imaging Modalities and Techniques

### 4.1. Ultrasound

US is increasingly acknowledged as a primary imaging technique for the brachial plexus, owing to its dynamic capabilities, superior spatial resolution, and cost-effectiveness. The patient is positioned either seated or supine with the neck extended and rotated contralaterally; the infraclavicular plexus is optimally examined with the arm in the ABER position. A conventional high-frequency linear transducer operates at 14–18 MHz, and ultra-high-frequency probes, ranging from 22 to 24 MHz, are advantageous for imaging small superficial nerves. Doppler and compression techniques facilitate the differentiation of nerves from arteries, while bilateral scanning enhances accuracy by employing symmetry as a reference [11].

A caudal-to-cranial scanning strategy assists in orientation. Scanning typically commences at the supraclavicular fossa, where the plexus manifests as a “cluster of grapes” posterolateral to the subclavian artery. From that point, trunks and cords can be followed distally into the axilla and proximally into the interscalene groove. The C5–C8 roots are located between the anterior and middle scalene muscles (C5 being the most superficial and C8 the deepest), but the T1 root, positioned beneath the first rib, necessitates meticulous medial angulation. Owing to the curvature of the cervical spine, the probe must be inclined anteriorly during cranial sweeps to preserve perpendicular alignment with the nerve. A typical peripheral nerve has a “honeycomb” configuration with hypoechoic fascicles surrounded by hyperechoic connective tissue in cross-section; in longitudinal view, it resembles electric wires with linear hyperechoic striations. This aids in distinguishing nerves from tendons or veins [12]. Ultrasound has been claimed to be as precise as MRI in identifying brachial plexus diseases, especially when performed by skilled practitioners [13]. An unfavorable scan clearly indicates the lack of substantial plexal disease, frequently rendering additional MRI unnecessary. US identifies anatomical variances (e.g., aberrant roots, auxiliary muscles) and is crucial for interventional planning, particularly in regional anesthesia. Real-time guidance enhances safety, mitigates the danger of vascular or nerve injury, and decreases procedure duration [14].

Clinical experiences suggest that US can reveal fascicular-level changes with better resolution than MRI, though this advantage requires skilled operators [15], and the diagnostic value of the fascicular disruption detection remains contingent also on lack of standard thresholds [6]. Similarly, recent high-resolution ultrasound studies have demonstrated the feasibility of morphometric assessment of the cervical vagus nerve, providing normative values for cross-sectional area (mean CSA: 2.3 mm^2^ on the right and 1.8 mm^2^ on the left) and fascicle count (mean: 2.4 right, 2.0 left), which can serve as reference for peripheral nerve evaluation in the cervical region [16].

Furthermore, recent advances in high-resolution ultrasound with matrix probes operating above 20 MHz have enabled reliable visualization of millimetric nerves, often below MRI detection thresholds, expanding the role of ultrasound in early neuropathy detection and interventional planning [17].

To summarize, ultrasound provides rapid, high spatial resolution and real-time dynamic evaluation of superficial nerves, with proven clinical value in evaluating long segments and identifying extrinsic compressions, in nerve sheath tumors, post-radiation fibrosis, metastatic infiltration, inflammatory neuropathies, and neuralgic amyotrophy. However, its limitations include incomplete visualization of the costoclavicular and retropectoralis spaces and inferior sensitivity compared to MRI [18]. MRI, conversely, offers superior soft-tissue contrast and early detection of muscle denervation: edema may appear within 24–48 h, whereas fatty atrophy reflects chronic denervation and is detectable months later, highlighting its role in both acute and chronic plexopathies [19].

#### Emerging US Tools

Advanced microflow techniques may improve the detection of intraneural microvascularity, aiding in the differentiation of inflammatory versus non-inflammatory neuropathies and enriching the evaluation of traumatic or post-radiation changes [20]. Contrast-enhanced ultrasonography (CEUS) offers real-time, dynamic assessment of intraneural perfusion, providing functional insights that complement conventional ultrasound and MRI. It may aid in differentiating benign from malignant lesions by analyzing microvascular patterns [21] and has shown intraoperative utility in schwannomas, MPNSTs, and margin delineation in complex neoplasms such as myxofibrosarcoma, especially when MRI is contraindicated [22]. CEUS outperforms power Doppler thanks to its superior signal-to-noise ratio and dynamic visualization of microvascular networks; however, its efficacy has been validated primarily in animal models, with limited human data mostly restricted to carpal tunnel syndrome [23].

Superb microvascular imaging (SMI) is another advanced ultrasound technique for detecting low-velocity flow in small vessels, showing improved performance over color and power Doppler. Yet, the current literature is sparse and primarily focused on carpal tunnel syndrome [23].

Ultrasound elastography (UE), including strain and shear wave elastography (SE, SWE), noninvasively evaluates tissue stiffness, potentially reflecting fibrosis or demyelination. While mainly studied in entrapment neuropathies, early findings support its use in the brachial plexus for assessing normative and pathological stiffness values [24]. SWE may also help evaluate nerve transfer outcomes via detection of muscle reinnervation. Nevertheless, elastography remains limited by significant interobserver variability and the lack of large-scale validation, particularly in brachial plexus disorders [25].

However, despite growing technical capabilities, the clinical implementation of CEUS, SMI, and elastography in brachial plexus imaging is still limited. This is primarily due to the absence of standardized acquisition protocols, the lack of normative databases, and the scarcity of large-scale studies confirming diagnostic thresholds and reproducibility.

### 4.2. Magnetic Resonance Imaging (MRI)

MRI is the primary modality for assessing the brachial plexus due to its exceptional soft-tissue contrast, multiplanar capabilities, and capacity to directly detect nerve morphology and adjacent structures. A well-designed procedure is crucial to navigate the intricate pathway of the plexus from the cervical spine to the axilla.

MR myelography using FIESTA sequences enables high-resolution, heavily T2-weighted imaging with excellent contrast between cerebrospinal fluid and nerve roots, allowing noninvasive visualization of preganglionic injuries; diffusion-weighted imaging (DWI), particularly with STIR-based diffusion-weighted neurography, offers selective depiction of postganglionic nerve fibers by exploiting their anisotropic water diffusion properties and can reveal discontinuities or signal loss in injured plexus segments [26]. 

High-field MRI with 3T devices is favored due to its superior signal-to-noise ratio (SNR) and contrast-to-noise ratio (CNR); however, a refined 1.5T technique is also permissible. Technical modifications—such as adjusting TR, TE, phase-encoding direction, and enhancing fat and blood suppression—can mitigate issues like air–shoulder interface artifacts and field inhomogeneity, resulting in high-quality visualization even at 1.5T [27]. Coil selection generally integrates posterior head-and-neck coils with adaptable surface coils to achieve extensive coverage. Bilateral imaging is advised to facilitate side-by-side comparison and identify slight asymmetries. Protocols must incorporate extensive field-of-view (FOV) coronal scans for a comprehensive overview and narrow FOV focused acquisitions for precise visualization. Coronal and sagittal scans must be oriented orthogonally to the nerve trajectory [28]. An exemplary basic protocol is presented in Table 2.

Fat-suppressed 3D T2-weighted sequences (e.g., IDEAL, STIR, Dixon) are essential for multiplanar nerve tracking and detecting edema, discontinuity, or mass effect. These sequences, referred to as MR Neurography, are most efficacious when utilized alongside oblique sagittal or coronal reconstructions [29].

#### Advanced MRI Techniques

Several sophisticated MRI methodologies augment diagnostic efficacy [30]: Diffusion Tensor Imaging (DTI) offers quantitative insights into nerve fiber integrity by evaluating anisotropic water diffusion. Parameters like fractional anisotropy (FA) and mean diffusivity (MD) are associated with axonal and myelin integrity. Avulsed nerve roots generally exhibit a reduction in fractional anisotropy and an elevation in mean diffusivity (MD), corroborating the diagnosis of traumatic brachial plexus injuries. Nonetheless, reproducibility is constrained for T1 roots owing to motion and anatomical variability.

Although DTI shows promise, there are technical limitations related to intraneural connective tissue and nerve angulation. Moreover, scan time constraints have historically limited its widespread clinical use, despite recent simplified vector models improving feasibility [31]. Furthermore, DTI quantitative metrics are strongly influenced by demographic variables such as age and BMI, limiting their generalizability across populations [32].

MR neurography enhances nerve visibility by attenuating adjacent fat, muscle, and vascular signals. High-resolution 2D and 3D sequences with isotropic voxels provide intricate multiplanar reconstructions that assist in monitoring nerve continuity and fascicular architecture, particularly in complex segments [33]. Plus, contrast-enhanced MR neurography integrates highly T2-weighted 3D STIR with gadolinium to enhance the visualization of nerve-tumor interactions, and it is especially useful for assessing neoplastic and treatment-associated plexopathies. Distinct indicators such as the “enhanced target sign” and “nerve tail sign” are linked to benign peripheral nerve sheath tumors (PNSTs), whereas nerve effacement or encasement indicates malignancy. Post-contrast delayed acquisitions enhance nerve-to-background contrast, facilitating a more assured evaluation of nerve-tumor interfaces and treatment strategies [34].

Recent pilot data demonstrated that low-dose ferumoxytol significantly improves vascular suppression and nerve-to-vessel contrast in 3D brachial plexus MR neurography compared to non-contrast techniques, without requiring additional flow-suppression gradients. Ferumoxytol may represent an alternative to gadolinium-based agents, particularly in patients with renal impairment, owing to its reticuloendothelial clearance and prolonged intravascular half-life, although larger clinical studies are warranted [35].

The interpretation of brachial plexus MR neurography is often limited by the complexity of the anatomy. Ontology-based interactive MR atlases [36] provide a standardized approach to anatomical recognition by linking imaging appearances to metadata (e.g., muscle innervation, entrapment sites, anatomic spaces), with potential educational and training benefits.

While US remains a dynamic and accessible modality, despite its highly operator-dependent accuracy, MR neurography provides superior soft-tissue contrast, but the lack of universally standardized MR neurography protocols across institutions may limit direct comparability of quantitative metrics [34]. 

Ultra-high-field 9.4T MRI has demonstrated near-perfect correlation with histological analysis for fascicle mapping, achieving submillimetric accuracy in number and morphology of fascicles, thus representing the current reference standard in ex vivo settings [37]. Although not yet applicable in clinical settings, these findings support the ongoing optimization of 3T MRI and AI-assisted neurography for fascicular resolution.

Resting-state fMRI (rs-fMRI) has been investigated as a method to evaluate cortical reorganization in individuals with traumatic injuries, uncovering modified connectivity between motor and sensory areas. It may offer predictive insights and assist in monitoring neuroplasticity during reconstruction [38].

Recent literature also points to the potential role of artificial intelligence in enhancing MRI interpretation, particularly through automated segmentation and radiomic-based prediction models [39]. Deep Learning (DL) reconstructions represent a novel frontier for enhancing picture quality while minimizing scan durations. DL methods can augment signal-to-noise ratio (SNR) in high-resolution 3D acquisitions, including rapid Dixon T2-weighted sequences, while preserving diagnostic quality without the need for contrast agents. This method mitigates artifacts associated with non-uniform fat suppression at elevated magnetic fields [40]. On another hand, DTI-derived metrics such as FA and MD, along with radiomic features, can distinguish between healthy controls and affected patients. However, these studies highlight the current limitations of these approaches, including small sample sizes, protocol variability, and the need for multicenter validation [41]. Despite its potential, AI-based methodology necessitates more validation across many platforms, scanners, and clinical settings prior to standard clinical implementation [42].

## 5. Diagnostic Patterns and Differential Diagnosis

### 5.1. Traumatic Plexopathy

Traumatic plexopathy is frequently caused by severe injuries, particularly in young adults following high-energy trauma such as motorcycle accidents or gunshot wounds, as well as in neonates with obstetric palsy, although the latter is not the focus of this research [9]. Epidemiologically, traumatic brachial plexus injury shows a strong male preponderance and is dominated by closed traction mechanisms. Supraclavicular segments (roots and trunks) are affected in roughly 90% of cases, and complete lesions occur in ≈53%, with upper elements more frequently involved than lower ones. Motorcycle crashes account for approximately two-thirds of traumatic presentations. Sports- and work-related mechanisms contribute substantially (~10%) to traumatic plexopathy, while winter sports account for ~4% of trauma-related cases. In contact sports, burners and stingers are common and likely under-reported due to rapid recovery, potentially masking the true incidence. Protective equipment (e.g., helmets) may shift injury patterns toward supraclavicular impacts [43]. Precise imaging is crucial for assessing the severity and scope of injury, distinguishing between preganglionic and postganglionic lesions, and informing surgical strategies. Traumatic lesions are clinically categorized as preganglionic, which involve the nerve root proximal to the dorsal root ganglion, or postganglionic, which impact trunks, divisions, cords, or terminal branches. This distinction is crucial: preganglionic injuries, like root avulsion, cannot be rectified with nerve grafts and generally necessitate nerve transfers; postganglionic injuries may be amenable to direct repair or grafting. Postganglionic nerve injuries are additionally categorized by the Seddon and Sunderland grading systems. Neurapraxia (Grade I) signifies a temporary conduction block while maintaining axonal integrity; neurotmesis (Grade V) denotes total nerve severance. Intermediate grades (II–IV) indicate escalating levels of axonal and fascicular damage, with Grade IV denoting a NIC. Neurography findings, as reported in Table 3, correspond accurately with these classifications: Grades II–III exhibit T2-hyperintense, enlarged nerves with obliterated fascicles and muscle denervation; Grade IV presents focal heterogeneous swelling with nerve injury complex development; Grade V indicates full discontinuity accompanied by end-bulb neuroma. Imaging should ideally be conducted 3–4 weeks after trauma to permit edema resolution and denervation manifestation [44].

In trauma, ultrasound is of limited value in the acute phase, but after 4–6 weeks, it can identify nerve discontinuity, stump neuromas, and perineural fibrosis, supporting surgical decision-making [18].

**Figure 3:**
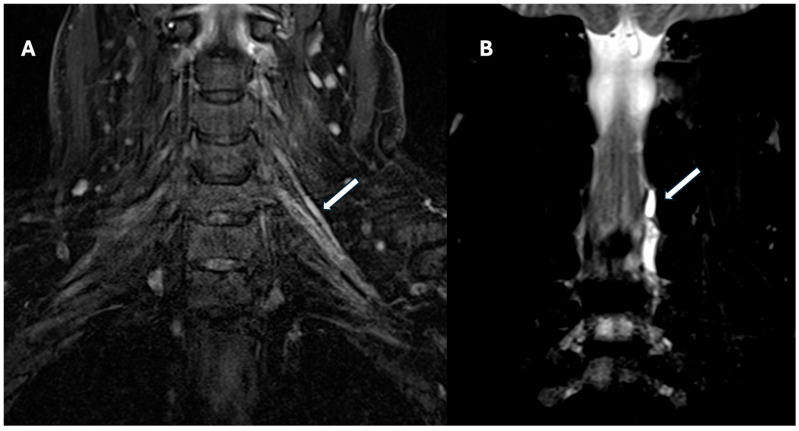
Coronal 3D STIR MR Neurography of the left brachial plexus. (**A**) Coronal 3D STIR image shows hyperintense signal of the left C5 and C6 nerve roots within the interscalene triangle (white arrow), extending into the upper trunk, consistent with postganglionic edema and axonotmesis. (**B**) Coronal T2-weighted turbo spin-echo (TSE) Dixon water-only image reveals multiple hyperintense pseudomeningoceles at the left neural foramina from C3–C4 to C5–C6 (white arrow), suggestive of dural tears and preganglionic root avulsion. These findings illustrate the coexistence of preganglionic and postganglionic features in traumatic brachial plexopathy.

MRI is the definitive standard for severe brachial plexus injury because of its multiplanar capabilities and superior soft tissue contrast. Extensive FOV coronal STIR or Dixon sequences offer a comprehensive picture, whereas limited FOV axial T2-weighted images are effective in identifying root avulsions, particularly adjacent to the dorsal root ganglia. Postganglionic injuries typically manifest as thicker nerves exhibiting low T1 and high T2 signal, occasionally accompanied by contrast enhancement. Alterations in signals from innervated muscles help pinpoint the location of the injury. Preganglionic injuries, especially root avulsions, are marked by root separation, pseudomeningoceles, and alterations in spinal cord signal (edema, hemorrhage, or myelomalacia) [9,45]. Pseudomeningoceles manifest as cerebrospinal fluid-filled outpouchings at neural foramina in up to 80% of avulsions; however, they are not pathognomonic. Indirect indicators are paraspinal muscle atrophy and enhancement of intradural root stumps. The T1 root has the most vulnerability, whilst the C5–C6 roots are more safeguarded; this is indicative of microanatomical variations, including a reduced number of fascicles and wider exit angles for anterior roots, as well as the influence of vascular supply (Figure 3). Novel methodologies, such as DTI, can provide corroborative evidence: avulsed roots have diminished FA and elevated radial diffusivity (RD). Nonetheless, repeatability continues to be constrained, particularly with T1 roots [46].

US is becoming increasingly significant for assessing terminal branches and superficial segments. It can surpass MRI in identifying postganglionic lesions, exhibiting a claimed sensitivity of up to 92%, and demonstrates a strong correlation with EMG findings, particularly for the ulnar and musculocutaneous nerves [28]. US can detect nerve swelling, discontinuity, hypoechoic neuromas, segmental enlargement, or hourglass constrictions that may not be seen on transverse MRI. It is beneficial for identifying direct compression from hematoma, callus, or bone fragments. For surgical planning, US aids in evaluating the suitability of postganglionic stumps for nerve grafting.

Effective care of traumatic brachial plexus injuries necessitates a multidisciplinary approach, integrating clinical assessment, electromyography (EMG), US, MRI, and microstructural techniques such as diffusion tensor imaging (DTI). Timely diagnosis—preferably within 4–6 weeks—is essential for facilitating nerve repair and enhancing functional results.

### 5.2. Neoplastic Plexopathy

Neoplastic involvement of the brachial plexus may arise from original neurogenic tumors, direct invasion by neighboring malignancies, metastases, or post-radiation alterations. Imaging is essential for distinguishing these entities, directing biopsies, and facilitating treatment planning. Primary neurogenic tumors including neurofibromas, schwannomas, plexiform neurofibromas, and malignant peripheral nerve sheath tumors (MPNSTs). Neurofibromas are prevalent, frequently plexiform in Neurofibromatosis type 1. Ultrasound generally reveals benign peripheral nerve sheath tumors as well-defined hypoechoic masses exhibiting posterior acoustic enhancement and maintained fascicular integrity. Ambiguous boundaries, osseous or pleural infiltration, or hypervascularity heighten the suspicion of malignancy. On MRI, schwannomas and isolated neurofibromas exhibit well-defined margins, isointensity on T1, hyperintensity on T2 (often presenting a “target sign”), and have pronounced post-contrast enhancement. Malignant peripheral nerve sheath tumors (MPNSTs) typically present as bigger masses with diverse T2 signal characteristics, uneven borders, and local infiltration, particularly in patients with neurofibromatosis type 1 (NF1) following radiation. Recent advancements in contrast-enhanced neurography facilitate the differentiation between benign and malignant tumors. Indicators like the “enhanced target sign” and “nerve tail sign” are characteristic of benign peripheral nerve sheath tumors (PNSTs), whereas nerve effacement or encasement implies malignancy [47]. Postponed CE-Neurography enhances tumor delineation. Metastatic plexopathy predominantly arises from breast cancer, succeeded by lung and head/neck malignancies. These tumors frequently affect lower trunks or cords as distinct, hypervascular masses with expansion to lymph nodes or the chest wall. The MRI reveals T1 hypointense and T2 hyperintense masses with significant enhancement, whereas CE-neurography aids in distinguishing the tumor from adjacent fibrosis. Pancoast tumors (apical lung malignancies) may infiltrate the brachial plexus through the interscalene triangle. MRI is crucial for early identification, since the loss of the interscalene fat pad signifies nerve invasion and potential surgical contraindication. Radiation-induced plexopathy generally manifests years post-therapy. MRI typically reveals widespread, symmetric T2 hyperintensity and modest enhancement without focal masses, suggesting fibrosis rather than recurrence. Asymmetric nodular growth accompanied by uneven enhancement indicates tumor recurrence. STIR or Dixon sequences enhance the identification of fibrotic tissue [2,3]. Ultrasound is excellent for demonstrating nerve enlargement, “dirty” perineural fat, and hypervascularity; infiltration manifests as segmental fusiform swelling, particularly in lower trunks [12]. A maintained epineural boundary indicates fibrosis instead of cancer.

### 5.3. Inflammatory and Iatrogenic Plexopathy

Inflammatory and iatrogenic plexopathies are diverse and might resemble neoplastic or traumatic lesions. US and neurography are crucial for precise diagnosis, lesion characterization, and monitoring.

Thoracic Outlet Syndrome (TOS) frequently arises from fibrous bands, cervical ribs, or atypical scalene insertions that compress the lower trunk. Ultrasound may reveal focal indentation or fusiform swelling (“wedge-sickle sign”), whereas dynamic Doppler can illustrate positional compression [12,20]. Neurography illustrates fibrous bands or vascular compression, particularly during arm abduction.

Neuralgic Amyotrophy (Parsonage–Turner Syndrome) is a critical mimic of traumatic or compressive plexopathy that frequently impacts the suprascapular nerve, though it may spread beyond the brachial plexus. Ultrasound reveals nerve hypertrophy, fascicular disarray, or hourglass constrictions [48].

MRI can demonstrate denervation-related T2 hyperintensity in the supraspinatus, infraspinatus, or deltoid muscles within a few days, whereas chronic atrophy and fatty replacement appear later [19]. Neurography may verify hourglass constrictions as fascicular narrowing without external compression, indicating an immune-mediated etiology [48]. Post-infectious PTS suprascapular nerve involvement further reinforces the association between immune-mediated triggers and selective brachial plexus neuropathies [49].

Chronic Inflammatory Demyelinating Polyradiculoneuropathy (CIDP) and Multifocal Motor Neuropathy (MMN) may affect plexus roots. US demonstrates symmetric or multifocal root enlargement accompanied by heightened intraneural flow; SMI can identify modest hypervascularity, which correlates with active inflammation and therapeutic response [3,20]. Quantitative thresholds have been established: a cross-sectional area > 8 mm^2^ in brachial plexus trunks, particularly when combined with enlargement of peripheral nerves, yields up to 99% specificity for chronic inflammatory neuropathies; a suprascapular nerve cross-sectional area > 4.2 mm^2^ demonstrates 86% sensitivity and 98% specificity for neuralgic amyotrophy [18].

Iatrogenic plexopathy may arise from surgical scarring, anesthetic damage, or catheter-associated trauma. US may indicate nerve discontinuity, localized edema, or fibrosis. Neurography reveals fascicular deformation, perineural scarring, or denervation atrophy [50].

## 6. Integrated Diagnostic Workflow

The integration of ultrasound and MRI is essential in cases with discrepant clinical and electrophysiologic findings. While US dynamically assesses compressive or post-traumatic lesions, MRI corroborates the presence and chronicity of denervation changes, thereby refining management decisions [19]. 

We propose a comprehensive comparative table (Table 4) that aligns the main brachial plexus pathologies—traumatic, neoplastic, inflammatory, and iatrogenic—with the most appropriate imaging techniques. This integration highlights the strengths, limitations, and ideal clinical indications of US, MRI, and a focus on CE-MR neurography advantages. Rather than fragmenting diagnostic performance into isolated sensitivity and specificity values, which may vary across settings, this framework emphasizes pragmatic applicability as a practical tool to support individualized, evidence-informed diagnostic workflows based on the specific clinical scenario and the anatomical level of involvement.

To summarize, US and MRI should be seen as complementary rather than redundant. US is an efficient first-line instrument owing to its accessibility, real-time evaluation, and good resolution for superficial nerves. It identifies fascicular disorganization, edema, hourglass constrictions, and heightened intraneural vascularity. Advanced modalities such as CEUS, elastography, and SMI can provide functional insights in specific, difficult instances. Nonetheless, intricate structures, preganglionic pathologies (e.g., root avulsions), or nuanced intraspinal abnormalities are more effectively assessed by MRI. 

US findings should always be linked with clinical assessment and electrodiagnostic evaluations; when US is normal, its negative predictive value (up to 97%) is high, and an MRI could be superfluous. Conversely, when problematic, MRI elucidates the magnitude, intraspinal involvement, or denervation not discernible on ultrasound.

Advanced methodologies such as DTI and contrast-enhanced MR neurography enhance specificity for complex situations such as concealed neoplasia or treatment-induced neuropathy [44,51]. Although neurography exhibits superior sensitivity for deep or intricate lesions, US may surpass MRI in evaluating superficial or terminal branches, allowing early visualization of fascicular disruption in superficial postganglionic lesions.

Recent validation studies show promising sensitivity relative to histological standards in early plexopathy scenarios, particularly where MRI may be unavailable or contraindicated, but while US shows high diagnostic value in detecting superficial fascicular disorganization, especially in early postganglionic injuries, its validation against histology remains limited to controlled settings and specific anatomical sites [37]. 

US and MRI have been widely employed in the evaluation of distal postganglionic neuropathies, such as ulnar nerve entrapment at the elbow, offering a valuable reference model for integrated nerve imaging. US normal appearance of the ulnar hyperechoic structure with a preserved fascicular pattern. In UNE, the nerve shows cross-sectional area (CSA) enlargement—typically exceeding 10 mm^2^ at the cubital tunnel—accompanied by hypoechogenicity, fascicular disruption, and increased intraneural vascularity on Doppler imaging. MRI, using fat-suppressed T2-weighted or STIR sequences, reveals nerve edema, thickening, and muscle denervation, supporting a three-tier morphological classification (signal alteration only, signal plus thickening, and associated muscle changes). The combined use of HRUS and MRI significantly improves diagnostic confidence and surgical planning and serves as a model for imaging strategies applicable to brachial plexus pathologies, particularly postganglionic segment evaluation [17].

The C6–C7 roots and the middle trunk are effectively evaluated by both modalities; however, the T1 roots are optimally analyzed with MRI due to acoustic shadowing. 

In clinical practice, US should be conducted prior to MRI, with neurography reserved for ambiguous or intricate lesions and always interpreted in conjunction with clinical and electrodiagnostic information. The integrated method guarantees an increased diagnostic yield.

In specific conditions such as post-traumatic NIC, US can depict segmental enlargement, hypoechoic structure, and loss of fascicular pattern, which may be sufficient for diagnosis and surgical planning, particularly when MRI is contraindicated or unavailable. Fascicular distortion, segmental swelling, or hourglass constrictions visualized on US may guide management in early postganglionic lesions. Although quantitative cutoffs are not standardized, fascicle disruption and hypoechogenicity extending over >2 cm may indicate significant injury warranting intervention, as supported by surgical correlations [7,37].

Prospectively, AI-driven instruments—such as deep learning reconstruction, feature-level fusion, and AI-enhanced compressed sensing—present significant advancements for modest non-traumatic plexopathies. Nonetheless, they necessitate rigorous multicenter validation and standardization prior to normal application [42]. AI-assisted compressed sensing (ACS) can effectively diminish scan durations while maintaining quality [52], and photon-counting detector computed tomography (PCD-CT) may serve as a complementary modality to MRI in specific instances, particularly where MRI is contraindicated [53].

## 7. Conclusions

The brachial plexus is among the most intricate and diagnostically demanding areas for radiologists. This review demonstrates that a systematic, cohesive methodology—incorporating high-resolution ultrasound and MRI, augmented by sophisticated techniques such as CEUS, elastography, SMI, DTI, and AI-assisted MR neurography—can markedly enhance diagnostic precision for traumatic, neoplastic, inflammatory, and iatrogenic plexopathies. Future prospective research and technical standardization will be crucial for validating these approaches and translating imaging breakthroughs into improved patient outcomes.

## Figures and Tables

**Table 1:** Anatomical Variants of the Brachial Plexus.

Variant Type	Description	Clinical Relevance	Approximate Incidence
Prefixed plexus	Large C4 contribution (C4–C8)	Higher risk of upper root injury	11% (6–17%)
Postfixed plexus	Involves T2 (C6–T2)	Risk of lower trunk avulsion	1% (0–1%)
Absent musculocutaneous nerve	Fibers travel with median nerve	Impacts surgical dissection, block strategy	~22.5%
Double musculocutaneous nerve	Two separate branches	May mimic neuroma/accessory branch	5–7%
Median–musculocutaneous communication	LeMinor types II–III common	Alters electrodiagnostic findings	5% (3–7%)
Crossing supraclavicular branch	Branch crosses over/lateral to clavicle	Important for interscalene block mapping	—

**Table 2:** Fundamental MRI Scanning Protocol.

Sequence	FOV (mm)	TR/TE (ms)	Primary Purpose	Practical Notes & Pitfalls
Coronal 3D STIR	~350	3000/194	Multiplanar reformats, global plexus trajectory.	Ensure robust and uniform fat suppression; useful for MIP reconstructions; motion artifacts can degrade quality.
Coronal T1-weighted	~320	647/6	Defines fat planes, anatomical borders, mass encasement.	Good for anatomical landmarks and fat infiltration; align with STIR for nerve-to-fat contrast correlation.
Axial STIR	~200	Variable	Targets C5–T1 roots & cords; detects edema, denervation.	Small FOV boosts spatial resolution; watch for magic angle effect near oblique fibers.
Axial T1-weighted	~200	Variable	Complements STIR for root morphology and pseudomeningoceles.	Best plane for subtle root avulsions or CSF leaks; compare with coronal T1 for continuity and side-to-side differences.
Sagittal T2-weighted	~240	4578/90	Evaluates cervicobrachial cord lesions, root continuity.	Good for root tracking from foramina to trunks; use in combo with coronal and axial; CSF pulsation can obscure detail.
Post-contrast T1 FS	—	Variable	Enhances nerve–mass interface, tumor infiltration, fibrosis.	Delayed acquisitions (CE-MRI) improve conspicuity; always compare with pre-contrast baseline; assess for enhancement.

**Table 3:** Seddon–Sunderland Classification and MR Neurography Patterns.

Grade	Classification	Definition	MR Neurography Findings	Treatment Implication
I	Neurapraxia	Temporary conduction block; axonal continuity intact	Normal or slight T2 hyperintensity; fascicles preserved	Spontaneous recovery
II	Axonotmesis	Axonal disruption; connective tissue sheaths intact	T2 hyperintense, nerve enlarged; loss of fascicular pattern; early muscle denervation	Possible spontaneous recovery; monitor
III	Advanced Axonotmesis	Axonal and partial fascicular damage	Marked T2 hyperintensity, fascicular disorganization; clear muscle denervation	Poorer prognosis; possible surgical repair
IV	Neuroma-in-Continuity (NIC)	Severe internal scarring within the nerve	Focal heterogeneous swelling; complex internal structure	Often requires surgical resection and nerve graft
V	Neurotmesis	Complete nerve transection	Complete discontinuity; end-bulb neuroma formation	Surgical repair or nerve grafting needed

**Table 4:** Comprehensive diagnostic workflow for brachial plexopathy.

Condition	US Findings	MRI Key Findings	CE-MRI Features	Distinguishing Clues
**Preganglionic Trauma**	Limited utility; deep root visualization is challenging	Pseudomeningoceles, root avulsion, spinal cord signal changes (edema, hemorrhage); root separation	Enhancement of intradural root stumps; better delineation of avulsed roots	Pseudomeningoceles (not pathognomonic), paraspinal muscle atrophy, most common at T1 root
**Postganglionic Trauma**	Swelling, discontinuity, neuroma, segmental enlargement, hourglass constrictions; highly sensitive for terminal branches	Thickened nerves, high T2 signal, neuroma-in-continuity, muscle denervation	May enhance neuromas and detect perineural fibrosis	Denervation in target muscles, correlation with EMG, useful for surgical planning
**Benign PNST**	Well-defined, hypoechoic mass, posterior enhancement, preserved nerve continuity	T1 iso, T2 hyper with target sign, intense enhancement	Target and tail signs	No invasion, mobile, ‘split-fat sign’
**MPNST**	Ill-defined, irregular, possible invasion	Heterogeneous T2, large, infiltrative	Nerve-effacing sign, no target	NF1, post-radiation, rapid growth
**Metastasis**	Discrete, hypervascular, lower trunks	T1 hypo, T2 hyper, enhances	Asymmetric, intense enhancement	Coexisting nodal disease
**Pancoast Tumor**	Limited	Apex mass invading triangle	Loss of interscalene fat pad	Shoulder pain, Horner’s, unresectable if fat pad lost
**Radiation Plexopathy**	Symmetric thickening, avascular	Diffuse symmetric T2 hyperintensity, mild enhancement	Homogeneous enhancement	Uniformity, late onset
**Post-Radiation Recurrence**	Irregular mass, possible vascularity	Focal, asymmetric, nodular enhancement	Asymmetric, nodular	Favors tumor if nonuniform
**Neuralgic Amyotrophy (PTS)**	Suprascapular nerve CSA > 4.2 mm^2^, fascicular disorganization, hourglass constriction	Hourglass constrictions, focal fascicular narrowing, muscle edema	—	CSA cutoff, dynamic fascicle changes
**CIDP/MMN**	Symmetric/multifocal root/median nerve enlargement, intraneural hypervascularity (SMI)	‘Onion bulb’ pattern in hereditary forms, diffuse root enlargement	—	Hypervascularity supports inflammation
**Thoracic Outlet Syndrome (TOS)**	Lower trunk indentation/swelling, ‘wedge-sickle sign’, dynamic Doppler with provocative maneuvers	Fibrous bands, vessel impingement, dynamic compression on positional MRI	—	Provocative maneuvers key for diagnosis
**Post-Surgical/Iatrogenic**	Nerve discontinuity, swelling, or perineural fibrosis at surgical site	Fascicular disruption, perineural scarring, muscle denervation	—	Post-surgical history, fibrosis pattern

## Data Availability

Not applicable.

## Abbreviations

The following abbreviations are used in this manuscript:

US	Ultrasound
MRI	Magnetic Resonance Imaging
MRN	Magnetic Resonance Neurography
CEUS	Contrast-Enhanced Ultrasound
SWE	Shear Wave Elastography
DTI	Diffusion Tensor Imaging
PNST	Peripheral Nerve Sheath Tumor
MPNST	Malignant Peripheral Nerve Sheath Tumor
TOS	Thoracic Outlet Syndrome
CIDP	Chronic Inflammatory Demyelinating Polyradiculoneuropathy
MMN	Multifocal Motor Neuropathy
EMG	Electromyography
SNR	Signal-to-Noise Ratio
CNR	Contrast-to-Noise Ratio
FA	Fractional Anisotropy
